# Treatment of HIV-Associated Lupus-like Membranous Nephropathy with Tacrolimus: A Case Report and Review of the Literature

**DOI:** 10.3390/life13030641

**Published:** 2023-02-25

**Authors:** Ioannis Kofotolios, Stathis Tsiakas, Chrysanthi Skalioti, Eleni Kapsia, George Liapis, Smaragdi Marinaki

**Affiliations:** 1 Department of Nephrology and Renal Transplantation, Laiko Hospital, Medical School, National and Kapodistrian University of Athens, 11527 Athens, Greece; 2 Department of Pathology, Medical School, National and Kapodistrian University of Athens, 11527 Athens, Greece

**Keywords:** glomerular diseases, HIVICK, HIVAN, HIV infection, lupus-like nephropathy, lupus nephritis, tacrolimus

## Abstract

Renal complications of HIV infection are common and histologically diverse. Besides HIV-associated nephropathy, which is the most well-defined glomerular disorder, immune-complex-mediated glomerulonephritis (HIVICK) is also encountered in the setting of HIV infection and may occasionally present with “lupus-like” features by light microscopy and immunofluorescence. Management of HIVICK remains controversial and mainly focuses on HIV viremia suppression with combined antiretroviral therapy. Immunosuppressive therapy may be used in order to mitigate the renal inflammation induced by the immune complex deposition. Data regarding the use of immunosuppressants in HIVICK are very limited, mostly including corticosteroids and mycophenolate acid analogues. Herein, we present the case of a 40-year-old HIV-infected Caucasian man with nephrotic syndrome, renal impairment, and a “lupus-like” membranous pattern in the kidney biopsy, who achieved a partial response of his proteinuria with a tacrolimus-based regimen in combination with antiretroviral therapy.

## 1. Introduction

A total of 38.4 million adults and children worldwide are currently living with human immunodeficiency virus (HIV) infection, according to the 2022 Joint United Nations Program on HIV/AIDS estimates [1]. Renal disease is an important cause of morbidity and mortality, affecting approximately 30% of HIV-positive patients [2,3]. A variety of renal manifestations have been described in patients with HIV infection, including HIV-associated nephropathy (HIVAN), immune complex glomerulonephritis (ICGN), thrombotic microangiopathy, and nephrotoxicity of antiretroviral therapy [4]. The introduction of highly active antiretroviral therapy (HAART) has resulted in increased life expectancy and a shift in the spectrum of renal diseases observed in HIV-infected patients (Table 1). Before the introduction of HAART, HIVAN was the leading cause of HIV-related renal disease and the most common cause of end-stage kidney disease (ESKD) in African American HIV patients. In the post-HAART era, sustained viral load suppression has led to a decline in the prevalence of HIVAN, while the incidence of ICGN and chronic kidney disease due to traditional risk factors, such as hypertension and diabetes mellitus (DM), has steadily increased [5,6].

ICGN in the setting of HIV infection (HIVICK) represents a heterogeneous group of glomerular diseases, such as IgA nephropathy, lupus-like proliferative glomerulonephritis, membranous nephropathy, and fibrillary glomerulonephritis [7]. Lupus-like nephritis is characterized by a “full-house” immunofluorescence pattern, defined by concurrent glomerular deposition of IgG, IgM, IgA immunoglobulins, C3, C1q complement components, in association with glomerular proliferative or membranous features by light microscopy. Extrarenal signs and symptoms of systemic lupus erythematosus (SLE), as well as serologic lupus antibody testing, are typically absent. [8].

Optimal treatment of patients with HIVICK is not yet established. Glucocorticoids have been used in patients with HIVAN with a favourable outcome [9,10,11,12]; however, data about immunosuppression therapy in HIVICK are limited. The use of HAART alone or in combination with immunosuppressive agents including mycophenolic acid analogues (MPAA) and glucocorticoids has been described in several cases with controversial results [13,14]. Furthermore, applying immunosuppressive therapy in already immunocompromised subjects raises concerns. Herein, we present a case of a patient with lupus-like nephropathy in the setting of HIV infection who was treated with tacrolimus and provide an overview of the current literature. 

## 2. Case Presentation

A 40-year-old Caucasian man with a history of HIV and hepatitis C virus (HCV) infection, associated with intravenous drug use, presented to the emergency department reporting lower extremity oedema that had progressively developed over the past month. HIV infection was diagnosed 11 years ago, when he first received HAART, albeit with poor compliance over the last 2 years. HCV infection had been successfully treated with pegylated interferon-a (IFN-alpha) and the viral load was undetectable for the past three years. The patient had reportedly quit intravenous drug use five years ago. 

At presentation, the patient’s blood pressure was 150/90 mmHg, the heart rate was 100 beats per minute, temperature 36.7 °C, respiratory rate 17 breaths per minute, and oxygen saturation was 98% on room air. Physical examination revealed mild bibasilar crackles, a palpable enlarged liver, and bilateral pretibial pitting oedema. There was no lymphadenopathy or skin rash. Chest radiography showed a small right pleural effusion and abdominal ultrasound showed liver enlargement (20 cm). 

On admission, the patient’s workup was remarkable for normocytic anemia (Hb: 9.7 g/dL), elevated ferritin levels (1.135 μg/L), polyclonal hypergammaglobulinemia (24 g/L), and a normal CRP value (Table 2). Renal indices showed mild kidney function impairment with a serum creatinine level of 99.1 μmol/L, corresponding to an estimated glomerular filtration rate (eGFR) of 70 mL/min per 1.73 m^2^ by CKD-EPI equation. He had nephrotic-range proteinuria (6.5 g/24 h) and a low serum albumin level (2.9 g/dL). Urine sediment examination revealed 25 to 30 erythrocytes of glomerular origin per high-power field. Ultrasonography displayed normal-sized kidneys. 

**Figure 1:**
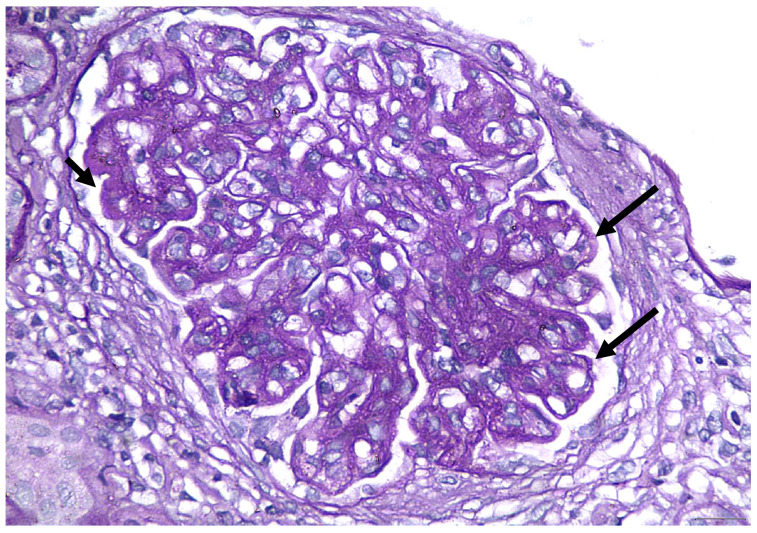
Light microscopy PAS stain X400: GBM thickening.

**Figure 2:**
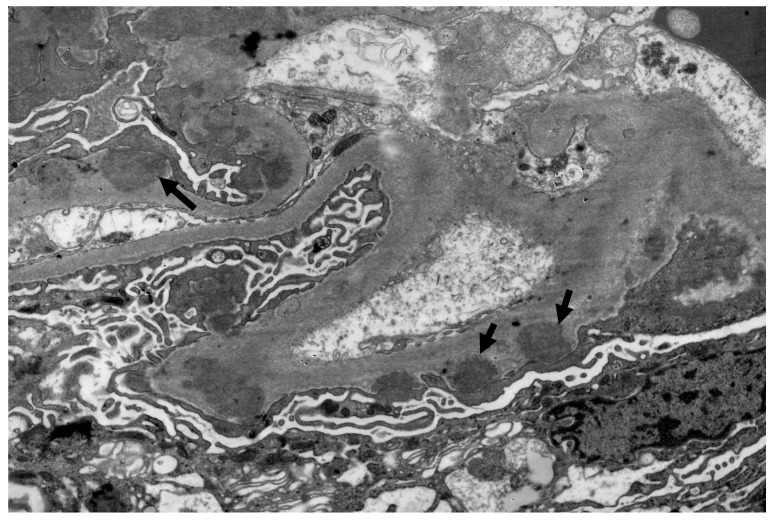
Electron microscopy: subepithelial deposits. (Uranyl acetate × 7100).

**Figure 3:**
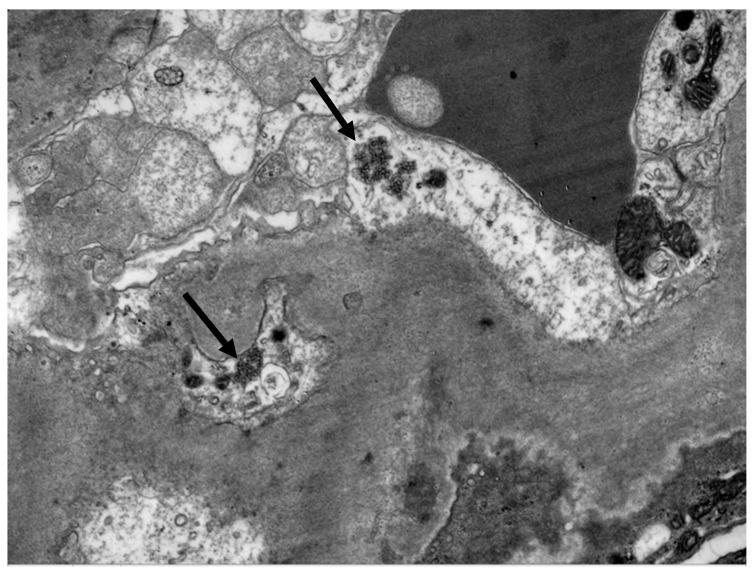
Electron microscopy: Tubuloreticular inclusions. (Uranyl acetate × 11,000).

A percutaneous renal biopsy was performed. Light microscopic examination revealed six glomeruli, two of which were globally sclerosed. Mild mesangial hypercellularity and diffuse thickening of the glomerular basement membrane (GBM) was noticed (Figure 1). In immunofluorescence examination, concomitant presence of IgG 3+, IgM 2+, IgA 1+, C3 3+, C1q 3+, kappa light chain 2+, and lambda light chain 3+ (0–3 + intensity scale) was observed, indicating a full-house pattern. The PLA2R staining was negative. Electron microscopy revealed numerous subepithelial and paramesangial deposits (Figure 2), as well as tubuloreticular inclusions (Figure 3). Histologic findings were compatible with a diagnosis of membranous nephropathy with lupus-like features.

Anti-nuclear antibodies (ANA), double-stranded DNA antibodies (anti-ds-DNA), antibodies to extractable nuclear antigens (ENA), and phospholipase A2 receptor antibodies (PLA2R) were negative. Serum C4 concentration was within the normal range, whereas a low serum C3 concentration was observed (69 mg/dL). Further testing showed a low CD4+ lymphocyte count of 66 cells/mm^3^ and an increased HIV RNA load of 3050 copies × 10^7^/mL.

In the absence of extrarenal and serological criteria of SLE, the diagnosis of lupus-like membranous nephropathy in the setting of HIV infection was made [15]. 

Combined antiretroviral therapy with tenofovir alafenamide, emtricitabine, and raltegravir was initiated. In addition, loop diuretics were added for oedema and co-trimoxazole for PCP prophylaxis due to immune deficiency. An ACE inhibitor was also initially administered, which was later discontinued due to an increase in serum creatinine levels (221 μmol/L). He remained on HAART for 2 months without renal response. Due to persistent nephrotic syndrome despite antiretroviral therapy, immunosuppressive treatment initiation was decided. Immunosuppressive therapy consisted of three intravenous methylprednisolone pulses of 500 mg, followed by oral prednisone (1 mg/kg/d), which was gradually tapered to 5 mg by Month 4 and mycophenolic sodium at an initial dose of 1080 mg/d and titrated to 1440 mg/d after two weeks. The patient was followed regularly at the renal department and HIV clinic. 

**Figure 4:**
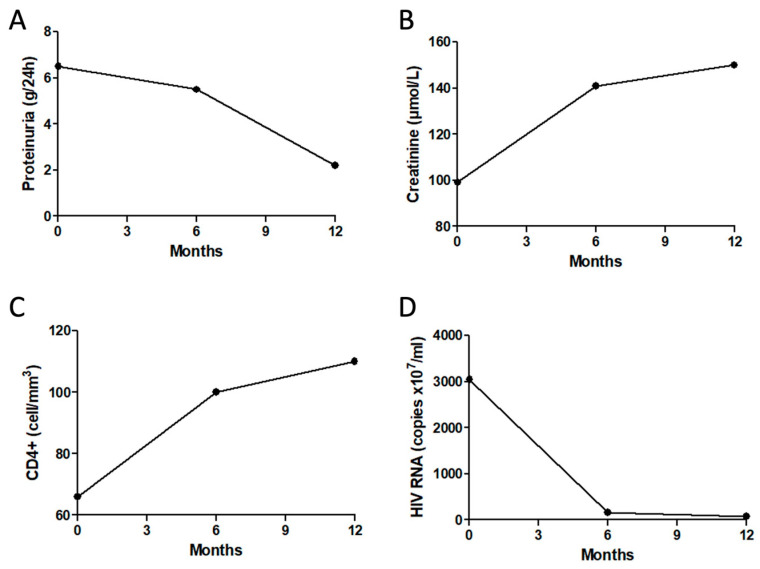
Line graphs showing (**A**) proteinuria (g/24 h), (**B**) creatinine (μmol/L), (**C**) CD4+ count (cell/mm^3^), and (**D**) HIV RNA viral load (copies × 10^7^/mL) at baseline and at 6 and 12 months after HAART treatment initiation.

Six months after treatment initiation, the CD4+ lymphocyte count had increased to 100/mm^3^, and the HIV viral load was undetectable (157 copies/mL). The patient’s nephrotic syndrome persisted, however, and no improvement in his kidney function was observed; serum creatinine levels were 141 μmol/L and urine protein levels were 5.5 g/d. Moreover, due to mycophenolate acid-related leukopenia development, mycophenolate sodium was discontinued. Based on previous experience with calcineurin inhibitors (CNIs) in nephrotic syndrome, particularly primary membranous nephropathy, we decided to initiate tacrolimus to control his proteinuria. The addition of tacrolimus to daily prednisone resulted in partial remission of his proteinuria after 12 months of treatment (2.2 g/d), with only a slight increase in serum creatinine levels (150 μmol/L) and no adverse events (Figure 4). 

## 3. Discussion

Renal dysfunction remains a frequent consequence of HIV infection, occurring in up to 30% of affected patients. Acute or chronic kidney disease may occur as a direct complication of HIV infection or as a result of combined antiretroviral therapy and other chronic comorbid conditions, including hypertension and type 2 diabetes mellitus. Despite the introduction of HAART, the prevalence of end-stage kidney disease (ESKD) continues to increase, affecting approximately 35% of patients with HIV-associated chronic kidney disease (CKD) [16]. On account of the increased risk for CKD among HIV patients, regular screening for renal impairment, especially in the African American population, is advised. Many potential reasons have been proposed, such as differences in patient preferences, environmental factors, and genetics. Acute kidney injury is present in 6% of hospitalized patients with HIV infection, and it is associated with a mortality rate of 27% [17,18]. Furthermore, HIV-infected patients may develop various glomerular disorders. The spectrum of glomerular lesions depends on race, accompanying infections, and response to HIV treatment [19].

Three HIV-associated histologic patterns of glomerular injury are more commonly observed: thrombotic microangiopathy (TMA), HIV immune complex kidney disease (HIVICK), and HIV-associated nephropathy (HIVAN). HIV-related thrombotic microangiopathy is associated with hematuria and proteinuria. The pathophysiological mechanism has not been fully elucidated but seems to be associated with ADAMTS-13 levels. [20,21]. Plasma exchange or plasma infusion appears to be an effective treatment option. The reported prevalence of HIVICK varies worldwide, which might, in part, reflect genetic differences worldwide. While HIVAN, a collapsing variant of focal segmental glomerulosclerosis (FSGS), is more commonly observed in African Americans patients, HIVICK seems to predominantly affect Caucasian and Asian populations according to recent studies [22]. Data from a large kidney biopsy series revealed that patients with HIVICK tend to have hypertension, increased CD4+ T cells, lower viral load, a higher estimated glomerular filtration rate, and a lower degree of proteinuria than HIVAN patients at the time of biopsy. They are also more likely to have hepatitis B or C virus coinfection and to use intravenous drugs [23]. Suppression of plasma viremia with HAART has been shown to improve kidney function in patients with HIVAN but does not appear to affect disease progression in HIVICK [24,25]. The spectrum of HIV nephropathy has changed with the introduction of HAART regimens. Notably, antiretroviral therapy can contribute to renal dysfunction either directly by inducing acute tubular necrosis, acute interstitial nephritis, crystal nephropathy, and renal tubular disorders or via drug interaction. Therefore, a systematic screening for kidney injury in all HIV patients on a regular basis is advised. However, potential renal side-effects of antiretroviral medication should not prevent clinicians from introducing HAART treatment in patients with kidney dysfunction, but close monitoring of renal function and early detection of risk factors are required.

HIV-associated lupus-like nephropathy is included in the spectrum of HIVICK and has been described in patients with renal biopsy features that share proliferative lupus nephritis features by light microscopy and immunofluorescence in the absence of clinical or serologic evidence of SLE. Light microscopic examination of renal tissue reveals a wide spectrum of findings including focal or diffuse mesangial hypercellularity and endocapillary proliferative lesions, fibrinoid necrosis and crescents, wire loops, spiky thickening of the glomerular basement membrane, tubular injury, microcysts filled with periodic acid-Schiff-positive casts, oedema, and inflammatory infiltrates of the interstitium. A “full-house” pattern is present on immunofluorescence, with concurrent IgG, IgM, IgA, C3, and C1q expression. Electron microscopy findings include large mesangial or subendothelial intra-membranous and subepithelial deposits and tubuloreticular inclusion bodies [26,27,28].

A literature search for relevant articles using PubMed, Medline, and Scopus was performed. No date limits were applied, and the last update was in January 2022. The keywords “lupus-like glomerulonephritis”, “lupus-like nephropathy”, “HIV-patients”, and “HIV-infection” were used. Search fields were restricted in the “Title/ abstract” and in human species. 

Since 1993, 83 patients with lupus-like nephritis and HIV infection have been reported worldwide (Table 3). In 1993, Nochy et al. reported that 22 of 60 (37%) French HIV-positive patients who underwent renal biopsy had some form of immune complex glomerulonephritis, with or without concurrent HIVAN, and 10 of these 22 biopsies showed histologic, immunofluorescence, and electron microscopic features resembling diffuse proliferative lupus nephritis [29]. In 1999, Casanova et al. reported the results of renal biopsies performed in 26 Italian HIV-positive patients, all Caucasian [30]. Twenty of these patients had immune complex glomerulonephritis, none had HIVAN. A total of 3 of the 20 cases with immune complex deposits showed proliferative glomerulonephritis with lupus-like features, with subendothelial deposits in all three cases and hyaline thrombi and “wire loops” in two cases, although immunofluorescence studies were not performed in one case. The largest and most recent cohort comes from Columbia University. It is a retrospective histopathologic analysis of all HIV-positive patients with kidney biopsy interpreted at Columbia University from 2010 to 2018 by Kudose et al. [22]. They reported 437 renal biopsies in HIV patients, identifying 59 cases of classic HIVAN, 66 HIVICK, and 9 lupus-like nephritis. To be noted, no data for treatment or clinical outcomes are reported in these case series. The first clinical outcomes for patients with HIVICK come from Haas et al. in 2005, who identified 14 cases of lupus-like glomerulonephritis among 77 renal biopsies in HIV-infected patients over a 5-year period in Baltimore. A poor one-year renal survival for 10 of the 14 patients was found [31]. In 2016, Booth et al. described a better prognosis for patients with HIVICK than HIVAN. During follow-up, 7.3% of patients with HIVICK versus 21.5% of patients with HIVAN died while 15% versus 52% developed ESKD [32]. Three cases with HIV lupus-like nephropathy among 263 total renal biopsies were also described in 2008 by Berliner et al., but this case series focused on HIVAN patients who were more likely to ultimately require hemodialysis (69.8% vs. 11.1% *p* < 0.0001) and had significantly worse survival than patients with HIVICK [33]. 

The optimal management of immune complex glomerulonephritis in the context of HIV infection is not clear. The role of immunosuppression has not been established yet. Single HIVICK cases, including patients with lupus-like features, where corticosteroids have been used, showed little benefit [34,35]. MPAA is the standard of care for induction and maintenance therapy in proliferative lupus nephritis with a favourable efficacy and tolerability profile. MPAA seems to modulate glomerular inflammation induced by immune complex deposition, thereby mitigating kidney injury. In HIVICK, HIV immune complexes circulating in the bloodstream are deposited in the kidneys. These circulating immune complexes trigger immune-mediated inflammatory kidney injury, which explains, in part, why HIV-associated glomerulonephritis shares similar histopathologic lesions with LN. These observations led Tiong et al. to suggest that patients with HIV-associated lupus-like nephritis may respond to therapies traditionally used in primary LN, which is why they were the first to use MPAA [13]. In line with the above, Kalyan et al. described the use of rituximab in an HIV patient with lupus-like membranous nephropathy who achieved partial remission [14]. Among the various case reports [36,37,38,39,40], two cases show interesting results in terms of therapeutic modalities. Matignon et al. [41], in 2005, and Yang et al. [39], in 2014, described two cases in which complete remission was achieved only using HAART therapy, while a case report by Chandran et al. [42] described a kidney transplant HIV patient with recurrent HIV-associated lupus-like nephritis whose kidney function has remained stable, even though no specific therapy was instituted. 

The benefit of calcineurin inhibitor (CNIs) in patients with nephrotic syndrome is well established. A dose-dependent increase in renal vascular resistance at the level of the afferent arteriole leading to a reduction in renal blood flow, glomerular filtration pressure, and ultimately the amount of albumin filtered is a principal mechanism for CNI-induced proteinuria reduction. Additionally, studies have shown that CNIs exert a protective effect on synaptopodin phosphorylation, which is critical for the integrity of the podocyte actin cytoskeleton. Considering the aforementioned studies, CNIs seem to stabilize the actin cytoskeleton, which is disrupted in patients with foot process effacement, improving the podocyte function [43,44,45,46,47]. Our patient presented with nephrotic syndrome and mild renal impairment in the setting of HIV infection. The renal biopsy revealed membranous nephropathy features with a “full-house” pattern by immunofluorescence. Based on the above literature for HIV-associated “lupus-like” nephropathy, we decided to treat him initially with HAART alone and, afterwards, in combination with MPAA and prednisone, with no improvement in his proteinuria. Considering the well-established antiproteinuric effect of CNIs, he was subsequently treated with tacrolimus, achieving partial remission of his proteinuria, and avoiding MPAA-associated adverse events.

## 4. Conclusions

This case highlights the important role of renal biopsy in HIV-infected patients for diagnosing and differentiating HIVICK from HIVAN and other glomerular diseases. No consensus for the management of HIVICK has yet been reported due to the limited existing data. The safety of immunosuppressive treatment in patients already at risk for opportunistic infections and the potential interactions with HAART raise concerns regarding the optimal treatment approach for HIV-associated immune complex glomerulonephritis. To our knowledge, this the first case of HIVICK that was treated with tacrolimus in combination with antiretroviral therapy. Based on our experience, calcineurin inhibitors may be considered as an additional safe therapeutic option in order to ameliorate proteinuria in patients with HIVICK. 

## Figures and Tables

**Table 1:** The spectrum of kidney biopsy findings in patients with HIV infection.

Diagnosis	
Podocytopathy	Classic HIVAN
	FSGS (NOS) in the setting of HIV
	Minimal change disease in the setting of HIV
	Other podocytopathy in the setting of HIV
Immune-complex-mediated glomerular disease	IgA nephropathy
	Uncharacterized ICGN with no etiology other than HIV IgG-dominant ICGN Lupus-like nephritis IgM-dominant ICGN IgG- and IgM-dominant ICGN Other ICGN NOS
	Membranous nephropathy
	Cryoglobulinemic GN
	Fibrillary GN
	Proliferative GN with monoclonal Ig deposits
Tubulointerstitial-dominant	Tenofovir nephrotoxicity
	Acute tubular injury
	Tubulointerstitial nephritis
	Chronic tubulointerstitial nephropathy NOS
	Acute tubular injury and/or tubulointerstitial nephritis
	IgG4-related disease
Vascular-dominant	Thrombotic microangiopathy
	Infarction
	Necrotizing arteritis

HIVAN: HIV-associated nephropathy, FSGS: Focal segmental glomerulorsclerosis, ICGN: Immunocomplex-mediated glomerulonephritis, NOS: Not otherwise specified, GN: Glomerulonephritis.

**Table 2:** Laboratory results.

	At Presentation	12 Months after Treatment	Normal Range
Serum			
Haemoglobin (g/L)	9.7	12.6	12–16
Leucocytes (×10^9^/L)	4.2	9.1	4.0–9.0
Thrombocytes (×10^9^/L)	168	289	120–360
Albumin (g/L)	2.9	4.2	3.5–5.0
CRP (mg/L)	0.94	1.3	<5
Creatinine (μmol/L)	99.1	150	45–83
eGFR (ml/min/1.73 m^2^)	70	58	>90
Triglycerides (mmol/L)	8.09	7.80	<1.7
Cholesterol (mmol/L)	12	9.8	<10
Ferritin (μg/L)	1.13	0.9	0.01–0.3
ANA	Negative	Negative	<1:80
Anti-dsDNA (IU/mL)	Negative	Negative	<7
ANCA	Negative	Negative	<20
Complement C3 (g/L)	69	111	90–180
Complement C4 (g/L)	24	22	10–40
CD4+ lymphocyte count (cell/mm^3^)	66	110	>470
HIV RNA (copies ×10^7^/mL)	3050	157	<50
Urine			
RBC	25–30	1–3	0–2
WBC	0–2	2–4	0–2
Protein (g/24 h)	6.5	2.2	<0.3

**Table 3:** Studies of kidney biopsy findings in HIV-positive patients.

First Author, Year	Country	Type of Study	Number of Patients	Results
Nochy D., 1993	France	Retrospective	60	HIVAN: 26, ICGN: 22; Lupus-like nephrits: 10, IgA: 4, TMA: 2, Interstitial-dominant lesions: 10
D’Agati V., 1998	United States	Retrospective	136	Lupus-like nephritis: 4, Other: 132
Casanova S., 1999	Italy	Retrospective	26	HIVAN: 0, Lupus-like nephritis: 3, IgAN: 4, Membranous nephropathy: 4, MCD: 2, Mesangial proliferative: 10, Crescentic GN: 1
Praditronsilpa K., 1999	Thailand	Retrospective	26	HIVAN: 0, Lupus-like nephritis: 0, Mesangial proliferative: 17, Membranous nephropathy: 2, Interstitial-dominant lesions: 2, IgAN: 2, Postinfectious GN: 3
Haas M., 2005	United States	Retrospective	77	HIVAN: 31, ICGN: 26, Lupus-like nephritis: 14, TMA: 1, Interstitial-dominant lesions: 4, Other: 15
Berliner AR., 2008	United States	Retrospective	152	HIVAN: 53, FSGS non-collapsing: 34, Interstitial-dominant lesions: 12, Postinfectious GN: 7, Lupus-like nephritis: 3, Other: 43
Nebuloni M., 2009	Italy	Retrospective	73	HIVAN: 9, ICGN: 40, Lupus-like nephritis: 5, IgAN: 4, Membranous nephropathy: 4, Other: 24
Foy M., 2013	United States	Retrospective	267	HIVAN: 56, ICGN: 83, Lupus-like nephritis: 11, IgAN: 8, Other: 122
Booth JW., 2016	United Kingdom	Retrospective	265	HIVAN: 70, ICGN: 92, Lupus-like nephritis: 10, IgAN: 27, Other: 103
Kudose S., 2020	United States	Retrospective	437	HIVAN: 119, ICGN: 75, Lupus-like nephritis: 9, Interstitial-dominant lesions: 113, TMA: 8, Other: 122

## Data Availability

The data that support the findings of this study are available from the corresponding author, upon reasonable request.

## References

[1] Global HIV and AIDS Statistics. https://www.unaids.org/en/resources/fact-sheet.

[2] Fine D., Wasser W., Estrella M., Atta M., Kuperman M., Shemer R., Rajasekaran A., Tzur S., Racusen L., Skorecki K. (2012). APOL1 risk variants predict histopathology and progression to ESRD in HIV-related kidney disease. J. Am. Soc. Nephrol..

[3] Fernando S., Finkelstein F., Moore B., Weissman S. (2008). Prevalence of chronic kidney disease in an urban HIV infected population. Am. J. Med. Sci..

[4] Wyatt C., Klotman P., D’Agati V. (2008). HIV-Associated Nephropathy: Clinical Presentation, Pathology, and Epidemiology in the Era of Antiretroviral Therapy. Semin. Nephrol..

[5] Rao T., Filippone E., Nicastri A., Landesman S., Frank E., Chen C., Friedman E. (1984). Associated Focal and Segmental Glomerulosclerosis in the Acquired Immunodeficiency Syndrome. N. Engl. J. Med..

[6] Bigé N., Lanternier F., Viard J., Kamgang P., Daugas E., Elie C., Jidar K., Walker-Combrouze F., Peraldi M., Isnard-Bagnis C. (2012). Presentation of HIV-associated nephropathy and outcome in HAART-treated patients. Nephrol. Dial. Transplant..

[7] Cohen S., Kimmel P. (2008). Immune Complex Renal Disease and Human Immunodeficiency Virus Infection. Semin. Nephrol..

[8] Maziad A., Torrealba J., Seikaly M., Hassler J., Hendricks A. (2017). Renal-Limited “lupus-Like” Nephritis: How Much of a Lupus. Case Rep. Nephrol. Dial..

[9] Smith M., Austen J., Carey J., Emancipator S., Herbener T., Gripshover B., Mbanefo C., Phinney M., Rahman M., Salata R. (1996). Prednisone Improves Renal Function and Proteinuria in Human Immunodeficiency Virus-associated Nephropathy. Am. J. Med..

[10] Sury K., Perazella M. (2019). The Changing Face of Human Immunodeficiency Virus-Mediated Kidney Disease. Adv. Chronic Kidney Dis..

[11] Yahaya I., Uthman O., Uthman M. (2013). Interventions for HIV-associated nephropathy. Cochrane Database Syst. Rev..

[12] Gupta S., Parker R., Robbins G., Dubé M. (2005). The effects of highly active antiretroviral therapy on albuminuria in HIV-infected persons: Results from a randomized trial. Nephrol. Dial. Transplant..

[13] Tiong M., Wilson S., Pham A., Chrysostomou A. (2020). Successful treatment of HIV-associated lupus-like glomerulonephritis with mycophenolic acid. Clin. Case Rep..

[14] Kalyan P., Schwartz D., Fisher M. (2021). Rituximab for Lupus-Like Membranous Nephropathy in the Setting of Well-Controlled HIV Infection. Am. J. Ther..

[15] Petri M., Orbai A., Alarcõn G., Gordon C., Merrill J., Fortin P., Bruce I., Isenberg D., Wallace D., Nived O. (2012). Derivation and validation of the systemic lupus international collaborating clinics classification criteria for systemic lupus erythematosus. Arthritis Rheum..

[16] Chaudhary S., Workeneh B., Montez-Rath M., Zolopa A., Klotman P., Winkelmayer W. (2015). Trends in the outcomes of end-stage renal disease secondary to human immunodeficiency virus-associated nephropathy. Nephrol. Dial. Transplant..

[17] Górriz J., Gutiérrez F., Trullas J., Arazo P., Arribas J., Barril G., Cervero M., Cofan F., Domingo P., Estrada V. (2014). Consensus document on the management of renal disease in HIV-infected patients. Nefrologia.

[18] Wyatt C., Arons R., Klotman P., Klotman M. (2006). Acute renal failure in hospitalized patients with HIV: Risk factors and impact on in-hospital mortality. AIDS.

[19] Nobakht E., Cohen S., Rosenberg A., Kimmel P. (2016). HIV-associated immune complex kidney disease. Nat. Rev. Nephrol..

[20] Ahmed S., Siddiqui R., Siddiqui A. (2002). HIV associated thrombotic microangiopathy. Postgrad. Med. J..

[21] Sarmiento M., Balcells M., Ramirez P. (2016). Thrombotic microangiopathy as first manifestation of acute human immunodeficiency virus infection: A case report and review of the literature. J. Med. Case Rep..

[22] Kudose S., Santoriello D., Bomback A., Stokes M., Batal I., Markowitz G., Wyatt C., D’Agati V. (2020). The spectrum of kidney biopsy findings in HIV-infected patients in the modern era. Kidney Int..

[23] George E., Nadkarni G., Estrella M., Lucas G., Sperati C., Atta M., Fine D. (2011). The impact of hepatitis C coinfection on kidney disease related to human immunodeficiency virus (HIV): A biopsy study. Medicine.

[24] Murphy R., Murray D., Robinson A., Campion B. (1989). Outcomes of cardiopulmonary resuscitation in the elderly. Ann. Intern. Med..

[25] Ifudu O., Sreepada T., Ao R., Tan C., Fleisch H., Chirgwin K., Friedman A. (1995). Clinical Study Zidovudine Is Beneficial in Human Im m unodeficiency Virus A ssociated Nephropathy. Am. J. Nephrol..

[26] Bajema I., Wilhelmus S., Alpers C., Bruijn J., Colvin R., Cook H., D’Agati V., Ferrario F., Haas M., Jennette J. (2018). Revision of the International Society of Nephrology/Renal Pathology Society classification for lupus nephritis: Clarification of definitions, and modified National Institutes of Health activity and chronicity indices. Kidney Int..

[27] Markowitz G., D’Agati V. (2007). The ISN/RPS 2003 classification of lupus nephritis: An assessment at 3 years. Kidney Int..

[28] Weening J., D’Agati V., Schwartz M., Seshan S., Alpers C., Appel G., Balow J., Bruijn J., Cook T., Ferrario F. (2004). The Classification of Glomerulonephritis in Systemic Lupus Erythematosus Revisited. J. Am. Soc. Nephrol..

[29] Nochy D., Glotz D., Dosquet P., Pruna A., Guettier C., Weiss L., Hinglais N., Idatte J.-M., Mery J.-P., Kazatchkine M. (1993). Nephrology Dialysis Transplantation Renal disease associated with HIV infection: A multicentric study of 60 patients from Paris hospitals. Nephrol. Dial. Transplant..

[30] Casanova S., Mazzucco G., Di Belgiojoso G.B., Motta M., Boldorini R., Genderini A., Monga G. (1995). Pattern of Glomerular Involvement in Human Immunodeficiency Virus-Infected Patients: An Italian Study. Am. J. Kidney Dis..

[31] Haas M., Kaul S., Eustace J. (2005). HIV-associated immune complex glomerulonephritis with “lupus-like” features: A clinicopathologic study of 14 cases. Kidney Int..

[32] Booth J., Hamzah L., Jose S., Horsfield C., O’Donnell P., McAdoo S., Kumar E., Turner-Stokes T., Khatib N., Das P. (2016). Clinical characteristics and outcomes of HIV-associated immune complex kidney disease. Nephrol. Dial. Transplant..

[33] Berliner A., Fine D., Lucas G., Rahman M., Racusen L., Scheel P., Atta M. (2008). Observations on a cohort of HIV-infected patients undergoing native renal biopsy. Am. J. Nephrol..

[34] Mialou V., Bertrand Y., Bouvier R., Nochy D., Fabien N., Nicoud P., Pondarré C., Meunier S., Philippe N., Cochat P. (2001). Lupus Nephritis in a Child with AIDS. Am. J. Kidney Dis..

[35] Yoon J., Chun S., Shin J., Lim B., Kim Y., Na K.-R., Suh K., Lee K., Shin Y.-T. (2009). A Case of Lupus-like Glomerulonephritis in an HIV-infected Patient. Infect. Chemother..

[36] Cook K., Stroup J., Stephens J. (2011). Newly Diagnosed White Man with HIV-Associated Lupus-like Nephropathy. Infect. Dis. Clin. Pract..

[37] Eanes G., Silva B., Leite V.A., Salgado-Filho N., Ravinal R., Gabriel J., Vergna G., Silva R., Dantas M. (2013). Necrotizing arteritis in a human immunodeficiency virus-infected patient with lupus-like glomerulonephritis. Int. J. Clin. Exp. Pathol..

[38] Hamid C., Hameed R., Khaliq B., Manzoor R., Hamid C. (2014). HIV associated lupus like nephropathy. Ethiop. J. Health Sci..

[39] Yang J., Seo M., Kim K., Lee J., Kim S.-C., Kim M.-G., Jo S.-K., Cho W.-Y., Kim H.-K., Won N. (2014). A case of lupus-like glomerulonephritis in an HIV patient with nephrotic range proteinuria, purpura, and elevated IgA level. Int. J. Clin. Exp. Pathol..

[40] Wiegersma J., Franssen C., Diepstra A. (2017). Nephrotic syndrome due to lupus-like glomerulonephritis in an HIV-positive patient. Neth. J. Med..

[41] Matignon M., Lidove O., Dupuis E., Walker F., Abgrall S., Papo T. (2005). A lupus-like glomerulonephritis following acute HIV-1 seroconversion in an African woman. Nephrol. Dial. Transplant..

[42] Chandran S., Jen K., Laszik Z. (2013). Recurrent HIV-associated immune complex glomerulonephritis with lupus-like features after kidney transplantation. Am. J. Kidney Dis..

[43] Malakasioti G., Iancu D., Tullus K. (2021). Calcineurin inhibitors in nephrotic syndrome secondary to podocyte gene mutations: A systematic review. Pediatr. Nephrol..

[44] Chen D., Jefferson B., Harvey S., Zheng K., Gartley C., Jacobs R., Thorner P. (2003). Cyclosporine A slows the progressive renal disease of Alport syndrome (X-linked hereditary nephritis): Results from a canine model. J. Am. Soc. Nephrol..

[45] Zietse R., Wenting G., Kramer P., Schalekamp M., Weimar W. (1992). Effects of cyclosporin A on glomerular barrier function in the nephrotic syndrome. Clin. Sci..

[46] Faul C., Donnelly M., Merscher-Gomez S., Chang Y., Franz S., Delfgaauw J., Chang J., Choi H., Campbell K., Kim K. (2008). The actin cytoskeleton of kidney podocytes is a direct target of the antiproteinuric effect of cyclosporine A. Nat. Med..

[47] Huber T., Kwoh C., Wu H., Asanuma K., Gödel M., Hartleben B., Blumer K., Miner J., Mundel P., Shaw A. (2006). Bigenic mouse models of focal segmental glomerulosclerosis involving pairwise interaction of CD2AP, Fyn, and synaptopodin. J. Clin. Investig..

